# Panic buying research: A bibliometric review

**DOI:** 10.3126/nje.v12i3.43436

**Published:** 2022-09-30

**Authors:** S.M. Yasir Arafat, Sujita Kumar Kar, Rakesh Singh, Vikas Menon, Brijesh Sathian, Russell Kabir

**Affiliations:** 1 Department of Psychiatry, Enam Medical College and Hospital, Dhaka-1340, Bangladesh; 2 Department of Psychiatry, King George’s Medical University, Lucknow-226003, U.P., India; 3 Department of Public Health, KIST Medical College, Tribhuvan University, Kathmandu, Nepal; 4 Department of Psychiatry, Jawaharlal Institute of Postgraduate Medical Education and Research (JIPMER), Puducherry-605006, India; 5 Geriatrics and long-term care department, Rumailah Hospital, Doha, Qatar; 6 School of Allied Health, Faculty of Health, Education, Medicine, and Social Care, Anglia Ruskin University, Chelmsford, United Kingdom

**Keywords:** Panic buying, bibliometric review, hoarding, perspectives, COVID-19, pandemic

## Abstract

**Background:**

Panic buying has been reported during a period of crisis when people buy an extra amount of essential commodities and hoard them anticipating their future utility. As a newer entity, a bibliometric analysis would reveal the research gaps for further studies. We aimed to do a bibliometric analysis of researches published on panic buying over the past two decades.

**Methods:**

A literature search was conducted in the SCOPUS database using the keyword “panic buying”. All published research in the English language between 1^st^ January 2001 to 1^st^ August 2021 was included in the analysis of this study.

**Results:**

We identified a total of 142 articles on panic buying published over the past two decades. There is an exponential increase in the publication on this topic during the COVID-19 pandemic (n=127). Majority of the articles were published from the United States (n=23), followed by the United Kingdom (n=20), and China (n=20). The *Frontiers in Public Health* and *Journal of Retailing and Consumer Services* published the highest number of articles (eight each). Arafat SMY published the highest number of publications as a single author (n=10) and *Enam Medical College and Hospital*, Bangladesh has the highest number of papers as an institution (n=10). Among all the publishers, Elsevier has published the maximum number of papers (n=38).

**Conclusion:**

There is an exponential growth of panic buying research during 2020-21. The global crisis of the COVID-19 pandemic has been attributed to the recent rise in panic buying research.

## Introduction

Panic buying is a behavioral event of buying things in larger quantities than usual, driven by fear and panic during the crisis period such as pandemics and public health emergencies [1,2]. Although panic buying has a longer history, it becomes a profound phenomenon recently during the COVID-19 pandemic [3-5]. Ever since the coronavirus disease (COVID-19) pandemic inflicted the world, countries across the globe adopted the public health measures including social distancing, travel restrictions, lockdown, quarantines, and use of personal protective equipment (PPE) for containing the disease transmission. Besides health, social and economic consequences of the pandemic on the daily life of individuals, it has also caused a change in consumer behavior. Stockpiling, aggressive in-store behaviors, compulsive hoarding, and shop raiding are some of the commonly observed consumer behaviors during the COVID-19 pandemic, worldwide [6,7].

There have been several reports of an drastic increase in purchases which occurred during the self-isolation period in the pandemic time that are uncharacteristic of the usual pre-pandemic time[8,9].Additionally, this conduct is common among people with high socioeconomic standing, which ultimately leads to shortages of necessities for people in lower socioeconomic classes. This inequity in the availability and accessibility of essential commodities such as food and medicines could make poor people more vulnerable to suffering. Therefore, it is very essential to study consumer anxiety affected by the COVID-19 pandemic leading to behavioral shifts and the measures imposed by different governments to contain the behavior of panic buying. Despite the growing understanding on the panic buying phenomenon, still there is lack of adequate scientific studies in this multi-dimensional field [3,10,11]. So, there is a need to draw evidence to understand this consumer behavior contextually to develop strategies for prevention and control of irrational panic buying. Although, this field is under-represented, as only few recent studies are published from limited researchers, worldwide. Therefore, it is very important for researchers to understand both perspectives i.e. various factors in changing consumer behavior, and changes in consumer behavior in health and socio-economic outcomes, in order to develop contextual country-specific strategies to prevent and control epidemics of panic buying. The gathered knowledge generated from the researches requires systematic conceptualization of the field of panic buying to determine the future directions of researches that will strengthen the scientific literature on precursors, and impact of panic buying.

During the pandemic, changes in consumer behavior and its consequences have led to the exploration of different facets of the panic buying literature. The identification of the main research themes in the panic buying literature especially during the pandemic can be done using the bibliometric analysis. Additionally, a discipline’s existing knowledge must be examined in order to advance an emergent study topic[12]. Bibliometric search enable the examination of past knowledge in any science field which handles the examination and evaluation of the scientific development [13,14]. It can reveal the existing problems in the field, help to understand and develop approaches for the elimination of the problems related to the concerned field. These methods also help to provide an overview of the academic research [15] of a scientific field or journal [16]. In addition, bibliometrics can help identify the contribution of publications from various countries, institutions, and authors [17]. Determining future research orientations by evaluating the studies on panic buying will contribute to the development of a broader perspective for the conceptualization of panic buying. However, to the best of our knowledge there has been no specific evaluation of panic buying based on bibliometric analysis in the literature. Henceforth, this study attempted to evaluate the publications related to “panic buying” using bibliometric methods to understand the bibliometric characteristics and research trends, globally. The study also evaluates the origin of the publication in terms of countries, organizations, authors, and journals.

## Methodology

### Search strategy

A literature search was performed in using *Scopus* database (www.scopus.com) for published articles on “panic buying” from January 01, 2001 till August 01, 2021with the search term as follows:


TITLE-ABS-KEY ("panic buying") AND PUBYEAR > 2001 AND (EXCLUDE (SRCTYPE, "p" ) OR EXCLUDE (SRCTYPE, "d" ) OR EXCLUDE (SRCTYPE, "b" ) OR EXCLUDE (SRCTYPE, "k" ) ) AND (EXCLUDE (DOCTYPE, "cp" ) ) AND (EXCLUDE (LANGUAGE, "Chinese" ) OR EXCLUDE (LANGUAGE, "Estonian" ) OR EXCLUDE (LANGUAGE, "Hungarian" ) )


### Inclusion criteria

We only included the articles published in the English language.

### Exclusion criteria

Conference proceedings, book chapters, articles published in other language [Hungarian (1), Estonin (1), Chinese (1)] and Trade journals were excluded.

#### Definition of panic buying

Panic buying is “the phenomenon of a sudden increase in buying of one or more essential goods in excess of regular need provoked by adversity, usually a disaster or an outbreak resulting in an imbalance between supply and demand” [2].

### Statistical analysis

Data were analyzed with help of the Microsoft Excel spreadsheet 2010 version. A simple descriptive statistic (frequency and percentages) was performed to depict the extraction.

### Permission

No formal ethical approval was sought as this bibliographic analysis reviewed the already published studies.

## Results

We found a total of 142 articles on panic buying from the SCOPUS database, published between 1st January 2001 to 1st August 2021. This included 119 original research articles, 10 review articles, 7 letters to editors (correspondences), 3 editorials, and 3 keynotes. Of the published articles, 55 (38.73%) resulted from international collaborative projects. The majority of the articles were published under the subject category of medicine (53), followed by business, management and accounting (32), social science (25), Economics, Econometrics and Finance (22), psychology (20), environmental science (10), Agricultural and Biological Sciences (9), Computer Science (8), Neuroscience (8), Energy (5), Immunology and Microbiology. The published articles were multidisciplinary involving Decision Sciences, Health Professions, Nursing, Arts and Humanities, Biochemistry, Genetics and Molecular Biology, Mathematics, Pharmacology, Toxicology and Pharmaceutics, Veterinary, Earth and Planetary Sciences, and Physics and Astronomy.

### Panic buying publications: Trend across time

There has been an exponential increase in the publications on panic buying during the COVID-19 pandemic. The highest frequency (more than half) of publications on panic buying has been documented in the year 2021, though the data till 1st August 2021 was considered in the analysis. In the years 2020 and 2021, (89.44%) of the articles on panic buying were published. The highest number of total citations (n=3056) and citations per paper (58.77) has been reported in the year 2020 (Table 1). International collaborative papers were highest in the year 2021 (n=30), followed by 2020 (n=23).

### Researchers of panic buying

A total of 159 authors had published articles on panic buying during the past 20 years. The number of papers published by the top 10 authors ranged from 3 to 10. A researcher from Bangladesh, *SM Yasir Arafat* is having the highest number of published papers (n=10) on panic buying who is also having the maximum number of total citations (n=147) and the international collaboration work (n=10) (Table 2). The highest number of citations per paper has been documented for the authors, Asmundson, G.J.G., Landry, C.A., and Paluszek, M.M., with 42 citations per paper (Table 2)

### Institutions contributing to panic buying research

A total of 160institutes / organizations contributed to panic buying research during the past 20 years. The number of papers published by the top ten institutes ranges between 3 to 10. The highest number of publications was from *Enam Medical College and Hospital*, Bangladesh. This institute is also having the highest number of total citations (n=147) and the highest number of international collaborations (n=10) (Table 3).

### Countries contributing to panic buying research

Over the past two decades, authors from 46 countries contributed to panic buying research. The number of publications on panic buying among the top ten countries ranges from 10 to 23. The highest number of publications (n=23) and international collaborations (n=16) were from the United States (US); but, the highest number of total citations is from the United Kingdom (n=1498). The highest number of citations per paper is from the Canada (CPP=104.20)(Table 4).

### Journals publishing panic buying research

The topic ‘*Panic buying*’ was published in 97journals during past two decades. The total number of papers published in the top ten journals ranges from 2 to 8. The highest frequency of papers (n=8) are published in the journal “Frontiers in Public Health” and “Journal of Retailing and Consumer Services”. The highest number of total citations had been documented in the papers published in Economics (n=222) followed by the Journal of Retailing and Consumer Services (n=192). The highest number of international collaborative work (n=5) in panic buying was published in the journal “Research and Public Health” (Table 5).

### Publishing houses publishing panic buying research

Most of the papers are published by the journals of Elsevier publishing house (n=38). The total number of publications by the top ten publishing houses range from 2 to 38. The highest number of total citations were from the articles published by the Elsevier group (n=2478) with the highest citations per paper (CPP=65.21). Most international collaborative works are published in the Elsevier group of journals (n=16) (Table 6).

## Discussion

The present article examined a total of 142 publications related to panic buying available on the SCOPUS database in the last two decades. These retrieved publications were originated from 22 disciplines (excluding multidisciplinary papers) that reinforce the transdisciplinary nature of the phenomenon of panic buying. Nevertheless, medical sciences and psychology contributed to more than half of the publications on panic buying (n=73, 51.4%) and only four multidisciplinary papers were identified. This indicates the need for greater collaborative research across disciplines to inform understanding and prevention of panic buying behavior.

Panic buying as a reactionary consumer behavior has a long history [1]. However, our findings clearly suggest that research on this phenomenon has proliferated exponentially following the onset of the COVID-19 pandemic. Nearly 90% of the research was published after 2020 with the highest number of citations (n=3056) and citations per paper (58.7) reported in 2020. This is suggestive of increasing research attention being paid to this phenomenon. The 142 publications originated from 46 countries; the highest number of publications emerged from United States (16.2%) followed by United Kingdom (15.5%), China, and India (14.1% each). These are also countries that were severely affected by the pandemic, and this may explain the increased research focus on panic buying in these nations.

The majority of the publications were original articles (83.8%) followed by review articles (7.0%), and correspondences (4.9%); other types of articles such as editorials and keynotes were relatively few (2.1% each). This is heartening and points to rigorous efforts being made to understand the phenomenon better. The relatively small number of reviews on panic buying may be reflective of limited original research on this topic; this can be expected to increase given the time trends in a proliferation of panic buying research. A little more than a third of papers published (n=55, 38.7%) involved collaboration between authors from different countries. Such papers are likely to inform cross-cultural perspectives on the phenomenon and must be encouraged.

A total of 159 authors contributed to global research output on panic buying in the last 20 years with the top 10 most productive authors contributing 33.1% of total research output and 31.9% of total citations. The fact that researchers from developing nations like Bangladesh and India constituted three of the five most productive authors on panic buying globally is intriguing suggests an upsurge of panic buying in these countries, possibly in the wake of the COVID-19 pandemic. A similar distribution was seen among the list of top ten most productive institutions. Given that the pandemic is likely to continue for few more years and similar future outbreaks, governments in these countries must incorporate findings from locally relevant research in their efforts to mitigate the impact of the pandemic.

The maximum number of papers were published in Frontiers in Public Health and Journal of Retailing and Consumer Services (n=8 in each); this was followed by the International Journal of Environmental Research and Public Health (n=7). This may indicate journal policies about prioritizing and fast-tracking papers related to panic buying in the wake of COVID-19. However, the higher number of citations per paper was found for articles published in Economics (111), Psychiatry Research (40), and British Journal of Health Psychology (23); this suggests that these journals published more impactful or quality papers than others. Global publishing houses such as Elsevier (26.8%) and Frontiers Media S.A. (10.6%) dominated panic buying research, probably owing to their wide collection of multi-disciplinary journals.

### Limitations and strengths

In spite of the fact that panic buying is not a new phenomenon, but it came to the attention of the scientific community during the COVID-19 pandemic. This is the first bibliographic review to assess the panic buying research from different institutions and countries. A good number of publications on this topic is recorded over a year and a half and this implies the significance and impact of this research topic. The review shares a skewed finding as a vast majority of the articles are published during the COVID-19 pandemic period and related to the pandemic. Hence, the matrices presented in this review mainly rely on the panic buying during the recent COVID-19 pandemic and this did not allow the researchers to calculate the citation account with the age of publication.

## Conclusion

The review identified a good number of publications on panic buying research published recently. The current review found that most of the publications on this topic are from the USA & UK and several articles published after 2020 received multiple citations. The key researchers who are actively publishing articles on panic buying research are from a leading institution in Bangladesh. Among the publishers, Elsevier and Frontiers are at top of the league in publishing panic buying research. Notably, a very high proportion of articles citation and collaborative publications has been observed in the first year of COVID-19 pandemic. This global crisis has been attributed to the sudden rise in the panic buying research. Furthermore, there is a pressing need for multidisciplinary collaborative research to better understand and develop mitigation strategies for panic buying behavior among general public.

## Figures and Tables

**Table 1:** Panic buying research- year wise output (n=142)

SN	Year	TP	TC	CPP	%TP	ICP	ICP%
1	2021	75	283	3.77	0.53	30	54.55
2	2020	52	3056	58.77	0.37	23	41.82
3	2019	3	29	9.67	0.02	0	0.00
4	2017	1	39	39	0.01	1	1.82
5	2016	1	1	1	0.01	0	0.00
6	2014	1	7	7	0.01	0	0.00
7	2013	1	4	4	0.01	0	0.00
8	2012	1	1	1	0.01	1	1.82
9	2011	1	0	0	0.01	0	0
10	2010	1	22	22	0.01	0	0
11	2008	1	0	0	0.01	0	0
12	2005	1	11	11	0.01	0	0
13	2003	1	10	10	0.01	0	0
14	2002	2	10	5	0.01	0	0

**TP- Total papers; TC-Total citations; CPP-Citations per paper; ICP-International collaborative paper**

**Table 2:** The top ten authors on panic buying research (n=142)

SN	Author	TP	TC	CPP	%TP	ICP	ICP%
**1**	Arafat, S.M.Y.	10	147	14.70	7.04	10	18.18
**2**	Kar, S.K.	8	140	17.50	5.63	8	14.55
**3**	Kabir, R.	5	133	26.60	3.52	5	9.09
**4**	Menon, V.	4	55	13.75	2.82	4	7.27
**5**	Taylor, S.	4	131	32.75	2.82	1	1.82
**6**	Yuen, K.F.	4	73	18.25	2.82	4	7.27
**7**	Asmundson, G.J.G.	3	126	42.00	2.11	1	1.82
**8**	Landry, C.A.	3	126	42.00	2.11	1	1.82
**9**	Paluszek, M.M.	3	126	42.00	2.11	1	1.82
**10**	Prentice, C.	3	52	17.33	2.11	1	1.82

**TP- Total papers; TC-Total citations; CPP-Citations per paper; ICP-International collaborative paper**

**Table 3:** The top ten institutions on panic buying research (n=142)

SN	Institute	TP	TC	CPP	%TP	ICP	ICP%
**1**	Enam Medical College and Hospital	10	147	14.70	7.04	10	18.18
**2**	King George's Medical University	8	140	17.50	5.63	8	14.55
**3**	Nanyang Technological University	6	74	12.33	4.23	5	9.09
**4**	Anglia Ruskin University	5	133	26.60	3.52	5	9.09
**5**	Jawaharlal Institute of Postgraduate Medical Education and Research	5	56	11.20	3.52	4	7.27
**6**	The University of British Columbia	4	131	32.75	2.82	1	1.82
**7**	North-West University	4	124	31.00	2.82	4	7.27
**8**	Griffith University	4	52	13.00	2.82	1	1.82
**9**	La Trobe University	3	45	15.00	2.11	3	5.45
**10**	University of Saskatchewan	3	224	74.67	2.11	1	1.82

**TP- Total papers; TC-Total citations; CPP-Citations per paper; ICP-International collaborative paper**

**Table 4:** The top ten countries on panic buying research (n=142)

SN	Country	TP	TC	CPP	%TP	ICP	ICP%
**1**	United States	23	991	43.09	16.20	16	29.09
**2**	United Kingdom	22	1498	68.09	15.49	14	25.45
**3**	China	20	914	45.70	14.08	12	21.82
**4**	India	20	213	10.65	14.08	11	20.00
**5**	Australia	16	167	10.44	11.27	7	12.73
**6**	Bangladesh	11	147	13.36	7.75	11	20.00
**7**	Germany	11	63	5.73	7.75	2	3.64
**8**	Singapore	11	824	74.91	7.75	9	16.36
**9**	Canada	10	1042	104.20	7.04	4	7.27
**10**	Indonesia	10	107	10.70	7.04	3	5.45

**TP- Total papers; TC-Total citations; CPP-Citations per paper; ICP-International collaborative paper**

**Table 5:** The top ten journals on panic buying research (n=142)

SN	Journal	TP	TC	CPP	%TP	ICP	ICP%
**1**	Frontiers in Public Health	8	22	2.75	5.63	4	7.27
**2**	Journal of Retailing and Consumer Services	8	192	24.00	5.63	3	5.45
**3**	International Journal of Environmental Research and Public Health	7	87	12.43	4.93	5	9.09
**4**	Frontiers in Psychiatry	3	8	2.67	2.11	1	1.82
**5**	Frontiers in Psychology	3	26	8.67	2.11	2	3.64
**6**	Global Business Review	3	5	1.67	2.11	0	0.00
**7**	Psychiatry Research	3	120	40.00	2.11	2	3.64
**8**	Sustainability	3	43	14.33	2.11	1	1.82
**9**	British Journal of Health Psychology	2	46	23.00	1.41	0	0
**10**	Canadian Journal of Agricultural Economics	2	222	111	1.41	0	0

**TP- Total papers; TC-Total citations; CPP-Citations per paper; ICP-International collaborative paper**

**Table 6:** The top ten publishers on panic buying research (n=142)

SN	Publisher	TP	TC	CPP	%TP	ICP	ICP%
**1**	Elsevier	38	2478	65.21	26.76	16	29.09
**2**	Frontiers Media S.A.	15	56	3.73	10.56	8	14.55
**3**	MDPI AG	13	141	10.85	9.15	8	14.55
**4**	Wiley	10	399	39.90	7.04	2	3.64
**5**	Emerald	9	68	7.56	6.34	3	5.45
**6**	SAGE Publications Inc.	9	88	9.78	6.34	3	5.45
**7**	Springer	10	63	6.30	7.04	4	7.27
**8**	Bellwether Publishing, Ltd.	3	8	2.67	2.11	1	1.82
**9**	Taylor and Francis	3	1	0.33	2.11	1	1.82
**10**	Dove Medical Press Ltd	2	6	3.00	1.41	1	1.82

**TP- Total papers; TC-Total citations; CPP-Citations per paper; ICP-International collaborative paper**
